# Prevalence and Antimicrobial Susceptibility of Bovine *Mycoplasma* Species in Egypt

**DOI:** 10.3390/biology11071083

**Published:** 2022-07-20

**Authors:** Ahmed M. Ammar, Marwa I. Abd El-Hamid, Yousreya H. Mohamed, Heba M. Mohamed, Dalal H. M. Al-khalifah, Wael N. Hozzein, Samy Selim, Wafaa M. El-Neshwy, Rania M. S. El-Malt

**Affiliations:** 1 Department of Microbiology, Faculty of Veterinary Medicine, Zagazig University, Zagazig 44519, Egypt; aaahmmad@vet.zu.edu.eg (A.M.A.); mero_micro@zu.edu.eg (M.I.A.E.-H.); h.moatz22@vet.zu.edu.eg (H.M.M.); 2 Department of Mycoplasma Research, Animal Health Research Institute, Agriculture Research Center, Giza 12622, Egypt; yousreya@ahri.gov.eg; 3 Department of Biology, College of Science, Princess Nourah Bint Abdulrahman University, P.O. Box 84428, Riyadh 11671, Saudi Arabia; dhalkalifah@pnu.edu.sa; 4 Botany and Microbiology Department, Faculty of Science, Beni-Suef University, Beni-Suef 62511, Egypt; hozzein29@yahoo.com; 5 Department of Clinical Laboratory Sciences, College of Applied Medical Sciences, Jouf University, Sakaka 72388, Saudi Arabia; sabdulsalam@ju.edu.sa; 6 Department of Animal Medicine, Infectious Diseases, Faculty of Veterinary Medicine, Zagazig University, Zagazig 44519, Egypt; wafaaesmail@zu.edu.eg; 7 Department of Bacteriology, Zagazig Branch, Animal Health Research Institute, Agriculture Research Center, Zagazig 44516, Egypt

**Keywords:** *Mycoplasm bovis*, cattle, fluoroquinolone resistance, *gyrA*, *parC*

## Abstract

**Simple Summary:**

Bovine *Mycoplasma* species, particularly antimicrobial resistant *Mycoplasma bovis* are important causes of bovine respiratory disease (BRD) in cattle, which causes major economic losses worldwide. Thus, the current study aimed to determine the prevalence and antimicrobial resistance profiles of bovine *Mycoplasma* spp. isolated from cattle’s respiratory tracts, in addition to evaluating the fluoroquinolone resistance in the recovered isolates using broth microdilution and conventional PCR techniques in Egypt. Our result showed that *M. bovis* was the most common spp. (61%), followed by *M. bovirhinis* (15%). In total, mycoplasma isolates were more prevalent among all examined lung tissues (38%), followed by nasal swabs (35%), tracheal tissues (28%), and tracheal swabs (27%). All the examined mycoplasma isolates (*n* = 76) were 100% susceptible to spectinomycin, tulathromycin, spiramycin, and tylosin, but high doxycycline and enrofloxacin minimum inhibitory concentrations (MICs) values were observed among 43.4% and 60.5% of the tested isolates, respectively. Three and two mycoplasma isolates with high enrofloxacin MICs were confirmed to be *M. bovis* and *M. bovirhinis*, respectively, by PCR assays. All molecularly confirmed mycoplasma isolates (*n* = 5) were positive for the *gyrA* gene (100%), meanwhile, three isolates (60%) were positive for the *parC* gene. In conclusion, understanding antimicrobial resistance mechanisms is a significant tool for the future development of genetic-based diagnostic techniques for the rapid detection of resistant mycoplasma strains.

**Abstract:**

Among many bovine *Mycoplasma* species (spp.), *Mycoplasma bovis* is recognized as a significant causative agent of respiratory diseases in cattle. In recent years, resistant *M. bovis* isolates, especially to fluoroquinolones, have been reported globally as a result of the extensive usage of antimicrobials in the treatment of bovine pneumonia. Therefore, the aim of this study is to investigate the prevalence and antimicrobial susceptibility patterns of bovine *Mycoplasma* spp. isolated from the respiratory tracts of cattle in Egypt and to assess the fluoroquinolones resistance in the recovered mycoplasma isolates via broth microdilution and conventional PCR techniques. Conventional phenotypic methods identified 128 mycoplasma isolates (32%) from 400 different samples, with *M. bovis* being the predominant spp. (61%), followed by *M. bovirhinis* (15%). Of note, mycoplasma isolates were rarely isolated from total healthy lung tissues (7/55, 12.7%), but they were frequently isolated from pneumonic lungs (31/45, 68.9%). All the examined mycoplasma isolates (*n* = 76) were sensitive to tilmicosin, tylosin, tulathromycin, spiramycin, and spectinomycin (100% each), while 60.5% and 43.4% of the examined isolates had high minimum inhibitory concentration (MIC) values to enrofloxacin and doxycycline, respectively. Three and two mycoplasma isolates with high enrofloxacin MICs were confirmed to be *M. bovis* and *M. bovirhinis*, respectively, by PCR assays. All molecularly confirmed mycoplasma isolates (*n* = 5) were positive for the *gyrA* gene (100%); meanwhile, three isolates (60%) were positive for the *parC* gene. In conclusion, our findings revealed alarming resistance to enrofloxacin and doxycycline antibiotics; thus, antimicrobial usage must be restricted and molecular techniques can help in the rapid detection of the resistant strains.

## 1. Introduction

*Mycoplasma bovis* (*M. bovis*) is a cell wall-less bacterium, which belongs to the class *Mollicutes* [1] and it is characterized by its small genome size and low G + C content (23–40%) [2]. It is considered to be a major cause of respiratory illnesses in young calves, and it is also considered to be one of the major causative agents of bovine respiratory disease (BRD), which is characterized by pneumonia in the form of low-grade fever, loss of appetite, mild depression, runny eyes, nasal discharge, dyspnea, hyperpnoea, and mild to continuous cough [3]. Additionally, *M. bovis* is considered the second most pathogenic bovine mycoplasma after *M. mycoides* subsp. *mycoides*, the causative agent of contagious bovine pleuropneumonia [4]. *Mycoplasma bovis* was first recognized from a case in the USA in 1961 [5], and since then, it has been correlated with a variety of clinical infections such as BRD, genital disorders, otitis media, mastitis, and arthritis [6]. *Mycoplasma bovis* is the most common species (spp.) isolated from cattle pneumonic lungs with case fatality rates as high as 45%. It can also persist in a herd for very long periods (a few weeks to several months) and it can be disseminated by the infected animals [7,8,9]. Healthy cattle exposed to *M. bovis* via the respiratory system may become chronic carriers but seldom develop diseases in the absence of stressors or other co-infecting microorganisms [10]. Of note, bovine pneumonic pasteurellosis, and bovine enzootic bronchopneumonia are caused by several organisms such as *Pasteurella* spp., *Haemophilus somnus, Actinomyces pyogenes,* and *M. bovis* may exacerbate the disease condition [10,11,12]. Moreover, mastitis due to *M. bovis* is a significant problem for milk production and animal welfare in large dairy herds around the world. It causes severe damage to the udder of cattle and it is largely untreatable by chemotherapy. The clinical signs include swelling and induration of the udder following a marked decrease in milk yield in dairy cows, calf mortality, and weight loss in surviving calves [6]. Furthermore, arthritis caused *M. bovis* mostly occurs in pre-weaned calves; however, it can occur in cattle at any age, and it is usually correlated with respiratory infection and it usually affects the big rotator joints (stifle, hock, shoulder, elbow, carpal, and hip). Additionally, the acute phase of the disease is characterized by pain, joint swelling, fever, and lameness with poor response to antimicrobial agents [10,12]. Otitis media interna in calves is believed to be caused by *M. bovis* either alone or in correlation with other microorganisms and it is characterized by poor appetite, conjunctival discharge, and pain with different degrees from pyrexia to neurologic manifestations [6,12].

Since no efficient vaccines are available, sanitary control measures and antimicrobial treatment remain the primary options for controlling *M. bovis* infections either as a preventive measure or in the early stages of the disease. Extended-spectrum fluoroquinolones such as marbofloxacin, enrofloxacin, and danofloxacin, long-lasting macrolides (tildipirosin, tulathromycin, and gamithromycin), florfenicol, and broad-spectrum cephalosporins (ceftiofur and cefquinome) are commonly used to treat or prevent BRD [13]. Additionally, *M. bovis* has recently become resistant to several antimicrobial classes such as fluoroquinolones, macrolides, and tetracyclines as a result of the uncontrolled usage of antimicrobial agents in the animal industry, which leads to economic losses due to treatment limitations by these antimicrobials [1].

Fluoroquinolones are the therapy of choice for a wide range of clinical and veterinary infections including *M. bovis* infection in cattle. Fluoroquinolones act via inhibiting the DNA gyrase and topoisomerases IV, which is fundamental for the replication of DNA [14]. Resistance to fluoroquinolone in many *M. bovis* strains is due to the chromosomal mutation in the quinolone resistance-determining regions (QRDRs) of the *parC* gene encoding topoisomerase IV and the *gyrA* gene encoding the DNA-gyrase, which is responsible for the high minimum inhibitory concentrations (MICs) of these antibiotics [15,16]. No alterations correlated to fluoroquinolone resistance have been reported so far in *parE* or *gyrB* genes in any clinical mycoplasma isolates. Meanwhile, two hot spots for mutations in *gyrA* and *parC* genes were reported for *M. bovis* isolates, which were responsible for the high minimum inhibitory concentrations (MICs) of these antibiotics [13].

Molecular techniques such as PCR are useful, rapid, and widely used for the detection of fluoroquinolone-resistant *M. bovis* strains using specific primers targeting DNA gyrase (*gyrA* gene) and/or topoisomerase IV (*parC* gene) [17,18,19]. Additionally, DNA sequencing is a direct and accurate method used for detecting the mutations in specific resistance genes such as *gyrA* and *parC*, which is important in understanding the antibiotic-resistant mechanisms that help in controlling the infection caused by fluoroquinolones resistant *M. bovis* strains [20]. Few reports have investigated the acquisition and the mechanisms of fluoroquinolone resistance in *M. bovis* in cattle [21]. Moreover, there is a lack of information about the antimicrobial resistance, especially to fluoroquinolones in bovine mycoplasma isolates in Egypt. Thus, the present work aimed to investigate the prevalence and the antimicrobial resistance patterns of bovine *Mycoplasma* spp. in the respiratory tracts of cattle, in addition to assessing the fluoroquinolone resistance in mycoplasma isolates via broth microdilution and conventional PCR techniques in Egypt.

## 2. Materials and Methods

### 2.1. Sample Collection and Study Area

In the current work, 100 animals of various sex, breeds, and ages (6–12 months old calves and 2–3 years old adult cattle) from three different abattoirs in Sharkia Governorate, Egypt, were sampled over a two-year period (August 2018–December 2020). A total of 400 samples (four different samples from each animal) were collected from apparently healthy (*n* = 55) and clinically diseased (*n* = 45) cattle, which all had respiratory symptoms including coughing, nasal discharges, abnormal lung sounds, with or without fever (Table 1). Before slaughtering, a clinical examination was performed to document age, sex, body condition scores, respiratory rate, and any obvious signs of the disease. After slaughtering, the animals were subjected to a post-mortem (PM) examination in accordance with the Egyptian guidelines for the inspection of cattle with special attention given to lung and tracheal tissues. Ethical approval was not required for the current work because all samples were obtained during routine diagnostic examinations and necropsies.

Among apparently healthy cattle (*n* = 55), 30 calves and 25 adult cattle were sampled. The collected samples (*n* = 220) comprised nasal and tracheal swabs and tracheal and lung tissues (*n* = 55 each). Moreover, among clinically diseased cattle (*n* = 45), 25 calves and 20 adult cattle were sampled and those animals showed respiratory symptoms (cough, abnormal lung sound, increased respiratory rate with or without fever), which are regarded as significant signs for mycoplasma infection. The collected samples (*n* = 180) included nasal and tracheal swabs and tracheal and lung tissues (multiple necrotic foci with caseous material and extensive fibrosis) (*n* = 45 each). Specimens were obtained under aseptic precautions utilizing a sterile scalpel to avoid surface contamination. Finally, the collected samples were transported to the laboratory within 2 h of sampling in special ice-filled containers.

### 2.2. Isolation and Identification of Mycoplasma Species

Mycoplasma isolation and identification were attempted from live and PM samples, which included materials from lung and trachea areas at the interface between healthy tissues and lesions [22]. Half a gram of each tissue sample was cut into small pieces and ground with sterile sand to be inoculated in pleuropneumonia-like organism (PPLO) broth (Oxoid, Basingstoke, UK) with mycoplasma-selective supplement G (Oxoid, Basingstoke, UK) as previously pronounced [23]. The PPLO agar (Oxoid, Basingstoke, UK) plates were inoculated with a loopful of the inoculated broth showing mycoplasma growth and the plates were incubated at 37 °C in microaerophilic humid conditions in an incubator with 10% CO_2_ and 95% N_2_ for 14 days, and the inoculated Petri dishes were examined every second day by stereomicroscope for determining the characteristic mycoplasma colonies (fried-egg appearance). Samples with no visible growth within the first week of inoculation were incubated until 21 days before being considered as mycoplasma negative. After that, the digitonin test was applied on the fried-egg-shaped colonies to differentiate among genus *Acholeplasma* and *Mycoplasma* utilizing dried filter paper discs impregnated with 0.2 mL of 1.5% ethanolic solution of digitonin. *Acholeplasma* spp. were digitonin resistant, while *Mycoplasma* spp. were sensitive [24]. Moreover, identification of *Mycoplasma* spp. was made by biochemical tests including arginine deamination, glucose fermentation, and film and spot formation [25]. Serological confirmation of *Mycoplasma* spp. was performed according to the procedures described previously [26]; meanwhile, species-specific identification was conducted using anti-*M. bovis*, anti-*M. bovirhinis*, anti-*M. bovigenitalium,* and anti-*M. arginini* hyperimmune sera via the growth inhibition methods using dried antisera impregnated paper discs [27].

### 2.3. Antimicrobial Susceptibility Testing via Broth Microdilution Method

Antimicrobial susceptibility procedure was conducted via broth microdilution technique [28] using customized 96-well microtiter plates (Trek Diagnostics, Independence, OH, USA) and eight antimicrobials representing five different antimicrobial classes approved for therapeutic applications in the veterinary field in Egypt. The used antimicrobials were fluoroquinolones (enrofloxacin (INVESA, Spain)), macrolides (tulathromycin (Zoeits, Canada), tilmicosin (ELANCO, Geneva, Switzerland), tylosin and spiramycin (ELANCO, Greenfield, IN, USA)), tetracyclines (doxycycline (Oxoid, Basingstoke, UK)), aminoglycosides (spectinomycin (Estonia, Poland)) and phenicols (florfenicol (Oxoid, Basingstoke, UK)). 

The MIC values of the antimicrobial agents against each isolate were determined by the broth microdilution method using PPLO broth medium (pH 7.8) (Difco™, BD Diagnostic Systems, Sparks, MD, USA) supplemented with 0.5% sodium pyruvate and 0.004% phenol red according to the method recommended by Hannan [28]. Briefly, each isolate was cultivated into 4 mL of the prepared broth and incubated aerobically for 48–72 h at 37 °C. A double-fold serial dilution was performed for each isolate utilizing the prepared broth with 200 μL total volume in each of the 12 wells. Ten microliters of the first six dilutions were cultivated onto the surface of PPLO agar plates (Difco™, Detroit, MI, USA) and the serial dilutions and the inoculated agar plates were incubated at 37 °C for 48–72 h with 80–100% relative humidity and 5–7% CO_2_ in the CO_2_ incubator. After incubation, the lowest dilution had a blue-red color change, and the colonies counted under stereomicroscope were used for determining the colony forming unit (CFU) and color-changing unit (CCU) counts of the isolates accordingly. Aliquots of mycoplasma isolates were prepared in PPLO broth to achieve 10^3^–10^5^ CCU/mL dilution. Double-fold serial dilutions of the tested antimicrobials were prepared and one-tenth milliliter of each antimicrobial dilution was added to 9 consecutive wells in the microtiter plates. MIC testing was performed by cultivating 200 μL of 10^3^–10^5^ CCU/mL of the mycoplasma isolate into every well of the microtiter plates. The microtiter plates were incubated for 24–72 h at 37 °C with 80–100% relative humidity and 5–7% CO_2_ until the positive control wells gave a blue-red color change. After that, the microtiter plates were read and any well that gave a blue-red color change was determined. The MIC was determined as the lowest concentration of the antimicrobial agent that completely inhibited the growth in the broth after one week (no color change). The CLSI has no approved MIC breakpoint values for the mycoplasmas of livestock [29]; thus, the results were interpreted according to previous publications [28,30,31,32] (Table 2). Additionally, MIC_50_ and MIC_90_ were determined as the lowest concentrations of the antimicrobial agents that inhibited 50% and 90% of the tested isolates, respectively [33].

### 2.4. Molecular Identification of Enrofloxacin-Resistant Mycoplasma Species by Conventional PCR and DNA Sequencing Techniques

#### 2.4.1. DNA Extraction

Total DNA extraction was performed as previously described [34]. Briefly, the bacterial lysates, which were utilized as templates for PCR assays were prepared via suspending a loopful of bacteria from a fresh overnight culture on a tryptic soy agar plate (Difco™, Detroit, MI, USA) in 200 μL of sterile water. After that, the mixture was boiled at 100 °C for five minutes to release the DNA, and then it was centrifuged. The supernatant was utilized as a template for all PCR assays.

#### 2.4.2. Conventional PCR Assays and Cycling Parameters

Three pairs of oligonucleotide primers were used in conventional PCR assays for amplification of *16S rRNA* genes of genus *Mycoplasma*, *M. bovis,* and *M. bovirhinis*. Additionally, another two primer pairs were used for the detection of the most important quinolone resistance-determining regions (QRDRs) genes (*gyrA* and *parC*). All PCR reactions were performed, in triplicate, using HotStarTaq Master Mix (Fermentanas, Thermoscientific, Waltham, MA, USA) following the manufacturer’s guidelines. The sequences of oligonucleotide primers utilized in all PCR assays are presented in Table 3. Agarose gel electrophoresis and ethidium bromide staining (Sigma, Burlington, MA, USA) for visualization of PCR products were performed as previously pronounced [35]. PCR-grade water (no template DNA) was utilized as a negative control, and reference strains of *M. bovis* (ATCC 25523) and *M. bovirhinis* (ATCC 27748) were utilized as positive controls in PCR assays.

### 2.5. Statistical Analysis

Our findings were analyzed utilizing SPSS Inc. version 26 (IBM Corp., Armonk, NY, USA). The Chi-square test was utilized to analyze the differences in the occurrence of *Mycoplasma* spp. from various origins and to assess the variations in the antimicrobial resistance rates of the obtained isolates from different sources and species. If *p* values were less than 0.05, they were recognized as statistically significant. All figures were performed via GraphPad Prism software version 8 (San Diego, CA, USA).

## 3. Results

### 3.1. Prevalence of Mycoplasma Species in Cattle

Regarding the phenotypic identification of *Mycoplasma* species, a total of 140 isolates (35%) from various 400 samples showed fried egg and/or centerless granular colonies on PPLO agar plates. Additionally, a total of 128 mycoplasma isolates (32%) representing multiple isolates from the same animal were digitonin positive, and they were identified phenotypically as the genus *Mycoplasma* (Table 4). Meanwhile, 12 isolates (3%) were digitonin negative and they were identified as the genus *Acholeplasma*; five were obtained from the nasal swabs of apparently healthy adult cattle and seven were obtained from the lung tissues of apparently healthy calves. Of note, a total of 76 mycoplasma isolates were obtained from 100 investigated animals (76%); each isolate represented a single animal.

**Figure 1:**
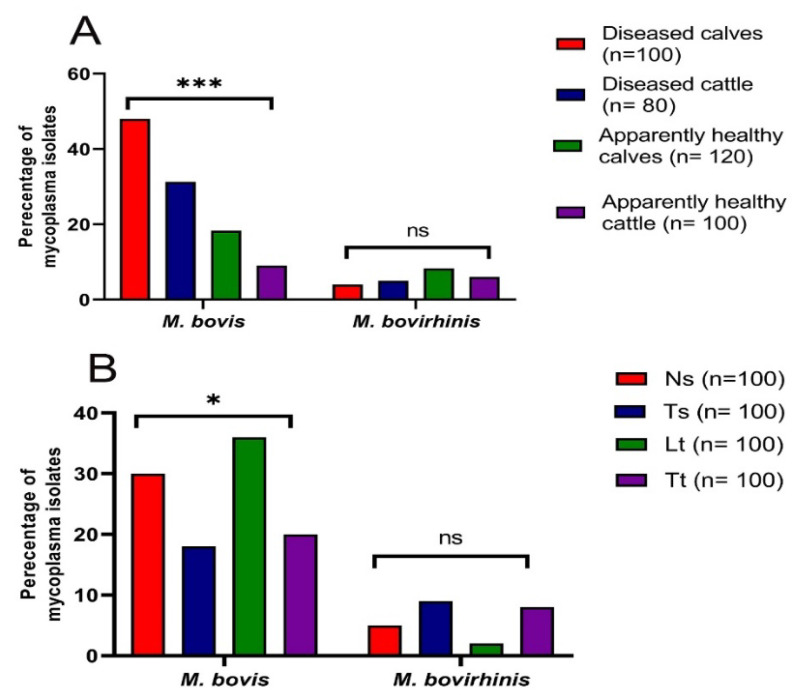
Prevalence of *M. bovis* and *M. bovirhinis* isolates in various samples’ sources (**A**) and types (**B**). Ns: nasal swabs, Ts: tracheal swabs, Lt: lung tissues; Tt: tracheal tissues, * *p* < 0.05, *** *p* < 0.001, ns: non-significant.

Considering the identification of multiple isolates from the same animal, *Mycoplasma* spp. were more prevalent among the collected samples from diseased calves (52%), followed by those from diseased cattle (36.3%) and apparently healthy calves and cattle (26.7 and 15%, respectively) (Figure 1A). Among the collected samples from apparently healthy calves and cattle, mycoplasma isolates were more prevalent among nasal swabs (30 and 24%, respectively), followed by tracheal tissues (26.7 and 20%, respectively) and tracheal swabs (26.7 and 16%, respectively), while lung tissues collected from apparently healthy cattle were mycoplasma negative. Among diseased calves and cattle, mycoplasma isolates were more prevalent among lung tissues (76 and 60%, respectively), followed by nasal swabs (48 and 40%, respectively), tracheal tissues (44 and 20%, respectively), and tracheal swabs (40 and 25%, respectively). In total, mycoplasma isolates were more prevalent among all examined lung tissues (38%), followed by nasal swabs (35%), tracheal tissues (28%), and tracheal swabs (27%) (Table 4 and Figure 1B). It was noted that mycoplasma isolates were rarely isolated from total healthy lung tissues (7/55, 12.7%), but they were frequently isolated from pneumonic lungs (31/45, 68.9%) (Table 4).

Regarding the identification of all mycoplasma isolates at the spp. level, *M. bovis* was the predominant spp. (61%), followed by *M. bovirhinis* (15%). *Mycoplasma bovis* was the predominant spp. among the collected samples from diseased calves (48%), followed by those from diseased cattle (31.3%) and apparently healthy calves and cattle (18.3 and 9%, respectively). Meanwhile, the highest isolation rates of *M. bovirhinis* were observed among the collected samples from apparently healthy calves (8.3%), followed by apparently healthy cattle (6%) and diseased cattle and calves (5 and 4%, respectively) (Figure 1A). The highest isolation rates of *M. bovis* isolates were observed among lung tissues (36%), followed by nasal swabs (30%), tracheal tissues (20%), and tracheal swabs (18%). On the other hand, the highest isolation rates of *M. bovirhinis* isolates were noticed among tracheal swabs (9%), followed by tracheal tissues (8%), nasal swabs (5%), and lung tissues (2%). Of note, highly statistical significance variations in the occurrence of total mycoplasma isolates and *M. bovis* were recognized among various samples’ sources (*p* ˂ 0.001); meanwhile, no statistically significant variations were recognized in the occurrence of *M. bovirhinis* among different samples’ sources (*p* = 0.59) (Figure 1A). Additionally, there were no statistically significant variations in the occurrence of total mycoplasma isolates and *M. bovirhinis* among various samples’ types (*p* = 0.28 and 0.16, respectively); however, there were statistically significant differences in the prevalence of *M. bovis* among different samples’ types (*p* = 0.01) (Figure 1B).

### 3.2. Antimicrobial Susceptibility Testing of Mycoplasma Isolates

Of the 128 recovered mycoplasma isolates, 76 were tested against 8 antibiotics by broth microdilution method for determination of their MICs. Those 76 isolates were randomly picked to represent all sample types and each isolate represented a single animal, and those isolates were recovered from apparently healthy calves (*n* = 20), apparently healthy cattle (*n* = 11), diseased calves (*n* = 25), and diseased cattle (*n* = 20) and each isolate represented a single animal. Interestingly, analysis of the antimicrobial susceptibility of the 76 examined isolates showed that all the tested isolates had low MICs values (sensitive) to tulathromycin, tilmicosin, tylosin, spiramycin, and spectinomycin (100% each), while 46 mycoplasma isolates (60.5%) had high MICs values (resistant) to enrofloxacin, and 33 mycoplasma isolates (43.4%) had high MICs values (resistant) to doxycycline (Table 5 and Table 6).

Regarding the species level of the recovered mycoplasma isolates, *M. bovis* isolates had high MICs values (resistant) to enrofloxacin (67.2%) and doxycycline (54.1%) antibiotics, while *M. bovirhinis* isolates had high MICs values (resistant) only to the enrofloxacin (33.3%) antibiotic (Table 6). There were statistically significant variations in the resistance rates of *M. bovis* and *M. bovirhinis* isolates against enrofloxacin (*p* = 0.01).

**Figure 2:**
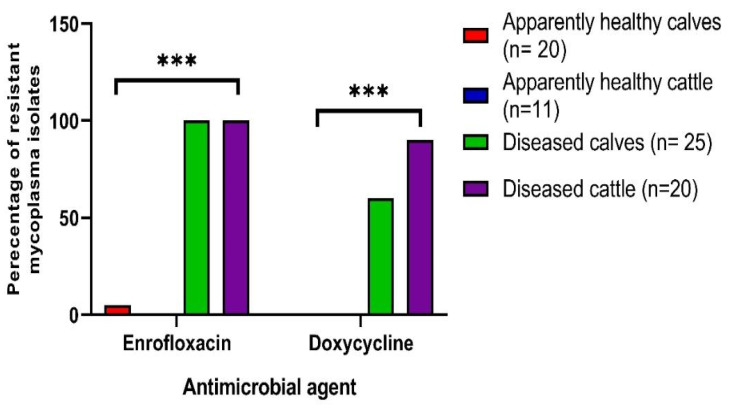
Prevalence rates of enrofloxacin and doxycycline among mycoplasma isolates from different samples’ sources. Ns: nasal swabs, Ts: tracheal swabs, Lt: lung tissue, Tt: tracheal tissue, *** *p* < 0.001.

Regarding the sources of the samples, high prevalence rates of enrofloxacin and doxycycline resistance were detected among mycoplasma isolates recovered from diseased calves (100 and 60%) and cattle (100 and 90%), respectively (Figure 2). There were highly statistically significant (*p* < 0.001) variations in the resistance rates of mycoplasma isolates from different samples’ sources against enrofloxacin and doxycycline (Figure 2).

### 3.3. Molecular Identification of Enrofloxacin-Resistant Mycoplasma Isolates from Different Sources

**Figure 3:**
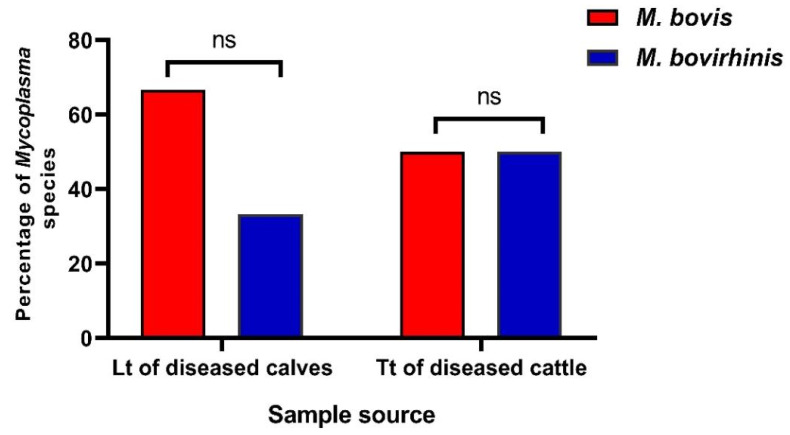
Prevalence of molecularly identified *Mycoplasma* species in the collected samples from different sources. The prevalence rate for each species was calculated regarding the total number of the examined samples from different sources. ns: non-significant, Lt: lung tissue, Tt: tracheal tissue.

Conventional PCR assays were carried out on five mycoplasma isolates with high enrofloxacin MIC values (≥4 µg/mL); three isolates were obtained from lung tissues of diseased calves and two isolates were obtained from tracheal tissues of diseased cattle. All the tested mycoplasma isolates (100%) were recognized as the genus *Mycoplasma* as they all harbored genes specific *16s rRNA* gene. Moreover, three isolates (60%) were confirmed to be *M. bovis*, while the remaining two isolates (40%) were confirmed to be *M. bovirhinis* as they were all positive for the presence of *16s rRNA* genes specific for each species (Figure 3). These findings were correlated with those of the phenotypic identification techniques. Of the three *M. bovis* isolates, two isolates were recovered from lung tissues of diseased calves, while the remained one isolate was obtained from the tracheal tissue of diseased cattle (Figure 3). Moreover, two *M. bovirhinis* isolates were obtained from lung tissue of a diseased calf and tracheal tissue of a diseased cattle (Figure 3). Moreover, no statistically significant variations were recognized in the occurrence of *M. bovis* and *M. bovirhinis* among different investigated samples’ types (*p* = 0.5 and 0.833, respectively) (Figure 3).

**Figure 4:**
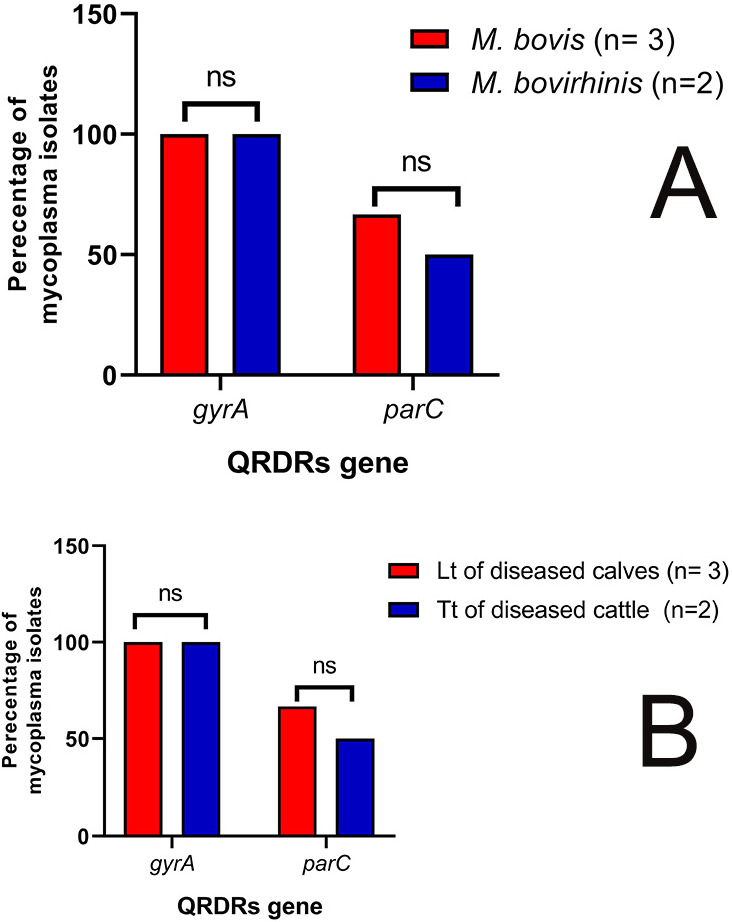
Prevalence of *gyrA* and *parC* genes in two bovine *Mycoplasma* species (**A**) and mycoplasma isolates from different samples’ sources (**B**). Lt: lung tissue, Tt: tracheal tissue, QRDRs: quinolone resistance-determining regions, ns: non-significant.

All molecularly confirmed mycoplasma isolates (*n* = 5) were examined for the presence of *gyrA* and *parC* genes of the quinolone resistance-determining regions. All the tested isolates were positive for the *gyrA* gene (100%). Meanwhile, three isolates (60%) were positive for the *parC* gene; two *M. bovis* isolates were recovered from lung tissues of diseased calves, and the one remaining *M. bovirhinis* isolate was recovered from tracheal tissue of diseased cattle (Figure 4). Of note, the *M. bovis* isolate with the highest enrofloxacin MIC value (8 ug/mL) was positive for both the *gyrA* and *parC* genes. Moreover, there were no statistically significant variations in the occurrence of the *parC* gene among mycoplasma isolates from different samples’ sources (*p* = 1) (Figure 4).

## 4. Discussion

It was pronounced that multiple global crises were emerging because of the extensive spreading of resistant bacteria such as *Mycoplasma* spp., *Klebsilla* spp., vancomycin-resistant *Staphylococcus Aureus,* and methicillin-resistant *Staphylococcus Aureus,* in addition to zoonotic foodborne bacteria such as *Salmonella Typhimurium*, *Salmonella Enteritdis,* and *Campylobacter* spp. [41,42,43,44,45,46,47,48,49,50,51,52,53]. The increase in the antimicrobial resistance rate of bovine *Mycoplasma* spp. to fluoroquinolone and tetracyclines is of great significance to the cattle industry globally. Herein, we determined a high prevalence rate of bovine *Mycoplasma* spp. (32%) among different samples’ types obtained from the respiratory tracts of cattle in Sharkia Governorate, Egypt. This result is partially similar to other studies conducted in Europe (34.3%) [54] and Turkey (35.4%) [55], and it was higher than the results of a previous study conducted in Nigeria (7.7%) [56] but lower than those results observed in other studies conducted in the USA (92%) [57] and Egypt (67.5%) [58]. Our findings demonstrated a high incidence of bovine *Mycoplasma* spp. recovered from lung tissues (38%). This result is partially similar to those of other studies conducted in Egypt, (40.9%) [59] and (36.8%) [60] and the USA (41.1%) [61], and it was higher than those recognized in previous studies conducted in Egypt, (31.66%) [62] and (29.6%) [63] but lower than those observed in other studies conducted in Canada (98%) [64], Denmark (86.8%) [65], the United Kingdom (86.4%) [66], and Argentina (70%) [67]. In the present study, bovine *Mycoplasma* spp. were more prevalent among lung tissues (38%) than nasal swabs (35%). This result is in complete agreement with those reported in previous studies conducted in Europe (60 and 24.4%, respectively) [54] and Canada (36 and 8.8%, respectively) [68], but it was in contrast with previous studies conducted in Europe, where *Mycoplasma* spp. were more prevalent among nasal swabs than lung tissues [69]. Herein, *Mycoplasma* spp. were more distributed among the samples collected from diseased calves (52%), followed by those obtained from diseased cattle (36.3%). These results were in complete agreement with those observed in recent studies conducted in Egypt (36 and 8.9%, respectively) [70] and Europe (17.1 and 11.4%, respectively) [54]. In the current study, *M. bovis* was the most predominant recovered spp. (61%) among the collected samples as documented previously in Iran (100%) [71], Britain (86.4%) [66], and Mexico (55%) [71,72]. Generally, the wide differences in the occurrence of bovine *Mycoplasma* spp. among different studies may be attributed to the environmental conditions, isolation and identification techniques, hygienic measures, types of the examined samples, animal breeds, time of sampling, management factors, stresses, and the geographical locations [70].

Recently, increased attention has been given to the significant role of *M. bovis* in BRD cases, which have become resistant to traditional antimicrobial therapy [29]. In the present study, 46 mycoplasma isolates (60.5%) were resistant to enrofloxacin with MICs values ranging from 2 to 8 μg/mL. These results were higher than those observed in other studies conducted in Europe (8.6%); (MICs ≥ 10 μg/mL) [73] and Spain (1.4%); (MICs ≤ 64 μg/mL) [74], but they were lower than those observed in a recent study conducted in Canada (100%); (MICs ≤ 128 μg/mL) [29]. Herein, all the tested mycoplasma isolates had low MICs values (sensitive) to macrolides (tilmicosin, tulathromycin, spiramycin, and tylosin); (100% each). This is in contrast with a recent study carried out in Switzerland, where high resistance rates (100%) were recorded against the macrolide group of antibiotics [75]. Moreover, all our examined mycoplasma isolates had low MICs values sensitive to spectinomycin. This is in contrast with a previous study conducted in Europe, which declared that 54.3% of the tested isolates had high MICs values (resistant) to spectinomycin [73]. Herein, mycoplasma isolates recovered from diseased cattle were more resistant to enrofloxacin (100%) than the apparently healthy ones. This result is in agreement with the findings of a previous study conducted in Canada, which reported that mycoplasma isolates from diseased cattle were more resistant to enrofloxacin (41.2%) than the healthy ones (23.3%) [29]. Additionally, doxycycline resistance was detected among 90% of our examined mycoplasma isolates that were recovered from diseased cattle; this result is in contrast with the findings of a previous study conducted in Israel, which reported that all the tested mycoplasma isolates were sensitive to doxycycline [32]. The high resistance rates of *M. bovis* isolates in developing countries may have resulted from the uncontrolled usage of antimicrobial agents in veterinary medicine as growth promoters, in cattle treatment in the case of respiratory diseases without any prescription, and as a control measure in farms, because there are no commercial vaccines available for bovine *Mycoplasma* spp. Therefore, antimicrobial usage must be controlled in the cattle industry [12]. Moreover, it is fundamental to utilize alternative drugs from medicinal plants to control resistant pathogens [76,77,78,79,80,81].

The PCR assays allow specific and quick detection of several bacterial pathogens associated with cattle pneumonia compared with the traditional isolation procedures [82]. In the present work, 60% of the tested isolates were identified as *M. bovis* and 40% were identified as *M. bovirhinis*. Interestingly, there was a 100% correlation between molecular and conventional identification results of the *Mycoplasma* species. These results are in agreement with a previous study conducted in Italy, where 70.3% of the tested isolates were identified as *M. bovis*, while 17.18% of the examined isolates were identified as *M. bovirhinis* [83]. Routine methods of using molecular techniques such as PCR and DNA sequencing can help in the rapid detection of the resistant mycoplasma strains. Thus, we recommend further large-scale studies to understand the different resistance mechanisms of fluoroquinolones and tetracyclines in mycoplasma strains obtained from different samples and animals of various health conditions.

## 5. Conclusions

Our findings revealed a high prevalence of bovine *Mycoplasma* spp. in the respiratory tract of cattle in Egypt. Moreover, our results showed alarming resistance to enrofloxacin and doxycycline in the examined mycoplasma isolates. Therefore, current antimicrobial usage rules must be followed to avoid an increase in antibiotic resistance, and fluoroquinolones and tetracyclines should be reserved for the treatment of serious diseases that have failed to respond to other antimicrobial classes. A major limitation in our approach is the need for DNA sequencing of *gyrA* and *parC* genes on large numbers of investigated isolates, in addition to fluoroquinolones sensitive and resistant strains, to understand the mechanism of fluoroquinolone resistance in bovine mycoplasma strains, which can help in the rapid detection of resistant mycoplasma strains and the management of mycoplasma infection.

## 6. Supplementary Aspect of Research

Sequencing of the PCR amplified products of *gyrA* and *parC* genes were conducted using an ABI 3730xl DNA sequencer (Thermo Fisher, Waltham, MA, USA). The amplified fragments were purified using the Gene Jet PCR purification kit (Cat. no. K0701, Thermoscientific, Waltham, MA, USA), and the sequencing of the purified products was performed with Big Dye Terminator V3.1 cycle sequencing kit (Applied Biosystems, PerkinElmer, Foster City, CA, USA) in an ABI 3130 automated DNA sequencer (Applied Biosystems, Carlsbad, CA, USA) according to the manufacturer’s guidelines. Sequence editing, consensus, and alignment construction were conducted utilizing BioEdit software package version 7.0.4.1. The phylogenetic trees were conducted utilizing MegAlign module for tree reconstruction of sequences by the Neighbor-joining technique based on ClustalW. Of note, the amino acid numbering was referred to *Escherichia coli* and it is based on the *E. coli* K-12 sequences for the *parC* (OM326889) and *gyrA* (OM326888) genes. The comparisons of obtained nucleotide sequences and multiple alignments were conducted utilizing the BioEdit sequence alignment editor (7.0.4.1) (Ibis bioscience, Inc., Carlsbad, CA, USA). Sequences of *gyrA* and *parC* genes were then submitted to NCBI GenBank utilizing BankIt (http://www.ncbi.nlm.nih.gov/WebSub/?tool=genbank) (accessed on 16 January 2022). under the accession numbers of OM326888 and OM326889, respectively.

One *M. bovis* isolate with high enrofloxacin MICs values (resistant) (MIC = 8 µg/mL), which was obtained from the lung tissue of a diseased calf, was subjected to DNA sequence analysis of *gyrA* and *parC* genes. Sequences of *gyrA* and *parC* genes of *M. bovis* isolate were submitted to the GeneBank database under the accession nos. of OM326888 and OM326889, respectively. The *gyrA* amino acid sequence of *M. bovis* isolate revealed 100% identity with those of other *M. bovis* isolates, *M. bovis* PG45 (reference strain; enrofloxacin sensitive), and *E. coli* K12 (Supplementary Figures S1–S3). Additionally, amino acids sequencing of the *parC* gene of *M. bovis* isolate revealed 100% identity when compared to other *M. bovis* isolates, and all these isolates showed 98% amino acid identity with *E. coli* K12 and 99% amino acid identity with *M. bovis* PG45 (control negative; enrofloxacin sensitive) (Supplementary Figures S4–S6). Additionally, the prediction protein of *parC* showed novel amino acid substitution at positions 2 (Gln→Arg); (CAG→CGT) in comparison with *M. bovis* PG45 (reference strain; enrofloxacin sensitive), as a first report in Egypt; thus, it might be considered the main cause of fluoroquinolone resistance in the examined isolate. This novel mutation is of interest and requires further scientific investigation on more isolates across the spectrum of fluoroquinolone susceptibility, and then a statistical inference is needed to show if the isolates that are resistant to fluoroquinolones are more statistically likely to have a mutation at position 2 than isolates that are susceptible.

Although fluoroquinolones are recognized to be effective antimicrobial agents in the treatment of bovine mycoplasmosis, mycoplasma isolates with high MICs (≤8 µg/mL) have been described in several countries [13]. Fluoroquinolone resistance usually develops as a result of target mutations in quinolone resistance-determining regions of the genes encoding gyrase and topoisomerases enzymes (*gyrA* and *parC*, respectively) [84]. In the current study, no amino acid substitutions were found within the quinolone resistance-determining regions of the *gyrA* gene, which is similar to a previous study carried out in France [13], but it was in contrast with a previous study conducted in Russia, where there was amino acid substitution in *gyrA* at positions 81 and 83 [85]. Additionally, a comparison of the *parC* quinolone resistance-determining regions in our tested isolates with the *M. bovis* PG45 strain (negative control; enrofloxacin sensitive) revealed the presence of amino acid substitutions at positions 2 (Gln→Arg), which, to the best of our knowledge, were described for the first time in Egypt; thus, it might be considered the main cause of fluoroquinolone resistance in the examined isolate. This result was consistent with previous studies conducted in several countries, which reported that fluoroquinolone resistance was due to mutations in the *parC* gene at positions 78, 80, 81, and 84 [85] and at position 84 [1,18]. Routine methods of using molecular techniques such as DNA sequencing can help in detecting mutations’ sites, which is important in understanding the resistance mechanism and can be helpful in the rapid detection of the resistant mycoplasma strains. Thus, we recommend further large-scale studies as an immediate future aspect of research to understand the different resistance mechanisms of fluoroquinolones and tetracyclines in *M. bovis* strains that have been obtained from different samples and animals of various health conditions.

## Figures and Tables

**Table 1:** Distribution of different samples collected from apparently healthy and diseased cattle in Sharkia Governorate, Egypt.

Sample Type (Symbol)	Animal Health Condition				Total
	Apparently Healthy (*n* = 55)		Diseased (*n* = 45)		
	Calve (*n* = 30)	Adult Cattle (*n* = 25)	Calve (*n* = 25)	Adult Cattle (*n* = 20)	
Nasal swab (Ns)	30	25	25	20	100
Tracheal swab (Ts)	30	25	25	20	100
Lung tissue (Lt)	30	25	25	20	100
Tracheal tissue (Tt)	30	25	25	20	100
Total	120	100	100	80	400

**Table 2:** Interpretative minimum inhibitory concentrations’ breakpoints of different antimicrobial agents against *Mycoplasma* species.

Antimicrobial Class	Antimicrobial Agent (Symbol)	MIC Breakpoint (µg/mL)			Reference
		Sensitive	Intermediate Sensitive	Resistant	
Fluroquinolones	Enrofloxacin (ENR)	≤0.25	0.5−1	≥2	[32]
Macrolides	Tulathromycin (TUL)	≤16	32	≥64	[31]
	Tilmicosin (TIL)	≤8	16	≥32	[32]
	Tylosin (TYL)	≤8	-	≥16	[28]
	Spiramycin (SP)	≤2−8	-	>8	
Tetracyclines	Doxycycline (DO)	≤1	2−4	≥8	
Aminoglycosides	Spectinomycin (SPT)	≤32	64	≥128	[30]
Phenicols	Florfenicol (FF)	≤2	4	≥8	

**Table 3:** Sequence of oligonucleotide primers and amplified PCR products for five target genes of *Mycoplasma* species.

Specificity (Target Gene)	Primer Sequence (5′-3′)	PCR Amplified Product (bp)	Reference
Genus *Mycoplasma* (*16S rRNA*)	F: AGACTCCTACGGGAGGCAGCA R: ACTAGCGAT TCCGACTTCATG	1000	[36]
*M. bovis* *(16S rRNA)*	F: CCTTTTAGATTGGGATAGCGGATG R: CCGTCAAGGTAGCATCATTTCCTAT	360	[34,37]
*M. bovirhinis* *(16S rRNA*)	F: GCTGATAGAGAGGTCTATCG R: ATTACTCGGGCAGTCTCC	316	[38,39]
QRDRs (*gyrA*)	F: GACGAATCATCTAGCGAG R: GCCTTCTAGCATCAAAGTAGC	531	[18,40]
QRDRs (*parC*)	F: GAGCAACAGTTAAACGATTTG R: GGCATAACAACTGGCTCTT	488	[18,40]

QRDRs: Quinolone resistance determining region, bp: base pair.

**Table 4:** Prevalence of bovine *Mycoplasma* species in various samples at Sharkia Governorate, Egypt.

Sample Type (Symbol, No.)	** Total No. of Mycoplasma Isolates from Various Samples of Investigated Animals (%)				Total *
	Diseased Calves (*n* = 100)	Diseased Cattle (*n* = 80)	Apparently Healthy Calves (*n* = 120)	Apparently Healthy Cattle (*n* = 100)	
Nasal swab (Ns, 100)	12 (48)	8 (40)	9 (30)	6 (24)	35 (35)
Tracheal swab (Ts, 100)	10 (40)	5 (25)	8 (26.7)	4 (16)	27 (27)
Lung tissue (Lt, 100)	19 (76)	12 (60)	7 (23.3)	0 (0)	38 (38)
Tracheal tissue (Tt, 100)	11 (44)	4 (20)	8 (26.7)	5 (20)	28 (28)
* Total (400)	52 (52)	29 (36.3)	32 (26.7)	15 (15)	128 (32)

* The isolation rates were calculated concerning the total number of the examined samples from each source and each animal. ** The isolation rates were calculated concerning the number of the samples from each animal.

**Table 5:** Minimal inhibitory concentrations of the tested antimicrobials against mycoplasma isolates.

Antimicrobial Class	Antimicrobial Agent (Symbol)	No. of Mycoplasma Isolates Showing MIC Values of the Tested Antimicrobials (μg/mL) *											MIC_50_	MIC_90_
		0.0078	0.0156	0.0312	0.0625	0.125	0.25	0.5	1	2	4	8		
Fluoroquinolones	Enrofloxacin (ENR)	-	-	-	-	-	5	10	15	41	4	1	2	2
Macrolides	Tulathromycin (TUL)	-	7	6	4	5	14	16	24	-	-	-	0.5	1
	Tilmicosin (TIL)	13	29	27	7	-	-	-	-	-	-	-	0.0156	0.0312
	Tylosin (TYL)	-	-	-	-	17	18	22	16		3	-	0.5	1
	Spiramycin (SP)	-	-	37	3	36	-	-	-	-	-	-	0.0625	0.125
Tetracyclines	Doxycycline (DO)	-	-	-	-	-	-	-	5	3	35	33	4	8
Aminoglycosides	Spectinomycin (SPT)	-	6	6	-	-	-	10	12	28	14	-	2	4
Phenicols	Florfenicol (FF)	-	-	-	-	-	-	6	26	17	27	-	2	4

* The dark vertical lines denote the resistance breakpoint, MIC: minimum inhibitory concentration, MIC_50_ = (*n* × 0.5), MIC_90_ = (*n* × 0.9).

**Table 6:** Antibiotic susceptibility patterns of total mycoplasma, *Mycoplasma bovis,* and *Mycoplasma bovirhinis* isolates.

		No. of *Mycoplasma* Species and Total Mycoplasma Isolates (%) Showing Susceptibility Patterns Against the Examined Antibiotic								
Antimicrobial Class	Antimicrobial Agent (Symbol)	*M. bovis* (*n* = 61)			*M. bovirhinis* (*n* = 15)			Total Mycoplasma Isolates (*n* = 76)		
		R	I	S	R	I	S	R	I	S
Fluoroquinolones	Enrofloxacin (ENR)	41 (67.2)	20 (32.8)	-	5 (33.3)	10 (66.7)	-	46 (60.5)	30 (39.5)	-
Macrolides	Tulathromycin (TUL)	-	-	61 (100)	-	-	15 (100)	-	-	76 (100)
	Tilmicosin (TIL)	-	-	61 (100)	-	-	15 (100)	-	-	76 (100)
	Tylosin (TYL)	-	-	61 (100)	-	-	15 (100)	-	-	76 (100)
	Spiramycin (SP)	-	-	61 (100)	-	-	15 (100)	-	-	76 (100)
Tetracyclines	Doxycycline (DO)	33 (54.1)	28 (45.9)	-	-	10 (66.7)	5 (33.3)	33 (43.4)	38 (50)	5 (6.6)
Aminoglycosides	Spectinomycin (SPT)	-	-	61 (100)	-	-	15 (100)	-	-	76 (100)
Phenicols	Florfenicol (FF)	-	21 (34.4)	40 (65.6)	-	10 (66.7)	5 (33.3)	-	31 (40.8)	45 (59.2)

R: resistant, I: intermediate sensitive, S: sensitive.

## Data Availability

Data sharing is not applicable to this article.

## Supplementary Materials

The following supporting information can be downloaded at: https://www.mdpi.com/article/10.3390/biology11071083/s1, Supplementary Figure S1. Phylogenetic tree of the examined *M. bovis* isolate based on *gyrA* sequence.; Supplementary Figure S2. Amino acid sequence alignments of *gyrA* gene of the examined *M. bovis* isolate.; Supplementary Figure S3. Amino acid identity percentages between *gyrA* genes of *E. coli* K12, *M. bovis gyrA* (OM326888), *M. bovis* PG45 (control negative; enrofloxacin sensitive), and other *M. bovis* isolates on GenBank.; Supplementary Figure S4. Phylogenetic tree of the examined *M. bovis* isolate based on *parC* sequence.; Supplementary Figure S5. Amino acid sequence alignments of *parC* gene of the examined *M. bovis* isolate.; Supplementary Figure S6. Amino acid identity percentages between *ParC* genes of *E. coli* K12, *M. bovis ParC* (OM326889), *M. bovis* PG45 (control negative; enrofloxacin sensitive), and other *M. bovis* isolates on GeneBank.
