# Supplementary material for: Prevalence and Antimicrobial Susceptibility of Bovine Mycoplasma Species in Egypt
Source: Biology (Basel). 2022 Jul 20;11(7):1083. doi: 10.3390/biology11071083 (PMC9312167; doi:10.3390/biology11071083)
Supplement: Supplementary file 1 [file biology-11-01083-s001.zip › biology-1766463-supplementary.pdf]

biology

MDPI

Article

Prevalence and Antimicrobial Susceptibility of Bovine *Mycoplasma* Species in Egypt

Ahmed M. Ammar [1,†], Marwa I. Abd El-Hamid [1,†], Yousreya H. Mohamed [2], Heba M. Mohamed [1], Dalal H. M. Al-khalifah [3], Wael N. Hozzein [4], Samy Selim [5], Wafaa M. El-Neshwy [6] and Rania M. S. El-Malt [7,*]

[1] Department of Microbiology, Faculty of Veterinary Medicine, Zagazig University, Zagazig 44519, Egypt; aaahmmad@vet.zu.edu.eg (A.M.A.); mero_micro@zu.edu.eg (M.I.A.E.-H.); h.moatz22@vet.zu.edu.eg (H.M.M.)
[2] Department of Mycoplasma Research, Animal Health Research Institute, Agriculture Research Center, Giza 12622, Egypt; yousreya@ahri.gov.eg
[3] Department of Biology, College of Science, Princess Nourah Bint Abdulrahman University, P.O. Box 84428, Riyadh 11671, Saudi Arabia; dhalkalifah@pnu.edu.sa
[4] Botany and Microbiology Department, Faculty of Science, Beni-Suef University, Beni-Suef 62511, Egypt; hozzein29@yahoo.com
[5] Department of Clinical Laboratory Sciences, College of Applied Medical Sciences, Jouf University, Sakaka 72388, Saudi Arabia; sabdulsalam@ju.edu.sa
[6] Department of Animal Medicine, Infectious Diseases, Faculty of Veterinary Medicine, Zagazig University, Zagazig 44519, Egypt; wafaaesmail@zu.edu.eg
[7] Department of Bacteriology, Zagazig Branch, Animal Health Research Institute, Agriculture Research Center, Zagazig 44516, Egypt
* Correspondence: rm.saeed@vet.zu.edu.eg
† These authors contributed equally to this work.

Citation: Ammar, A.M.; Abd El-Hamid, M.I.; Mohamed, Y.H.; Mohamed, H.M.; Al-khalifah, D.H.M.; Hozzein, W.N.; Selim, S.; El-Neshwy, W.M.; El-Malt, R.M.S. Prevalence and Antimicrobial Susceptibility of Bovine *Mycoplasma* Species in Egypt. *Biology* **2022**, *11*, 1083. https://doi.org/10.3390/biology11071083

Academic Editor: Chrissoula Voidarou, Athina S. Tzora, Georgios Rozos and Huizhong Chen

Received: 26 May 2022
Accepted: 15 July 2022
Published: 20 July 2022

Publisher's Note: MDPI stays neutral with regard to jurisdictional claims in published maps and institutional affiliations.

Copyright: © 2022 by the authors. Licensee MDPI, Basel, Switzerland. This article is an open access article distributed under the terms and conditions of the Creative Commons Attribution (CC BY) license (https://creativecommons.org/licenses/by/4.0/).

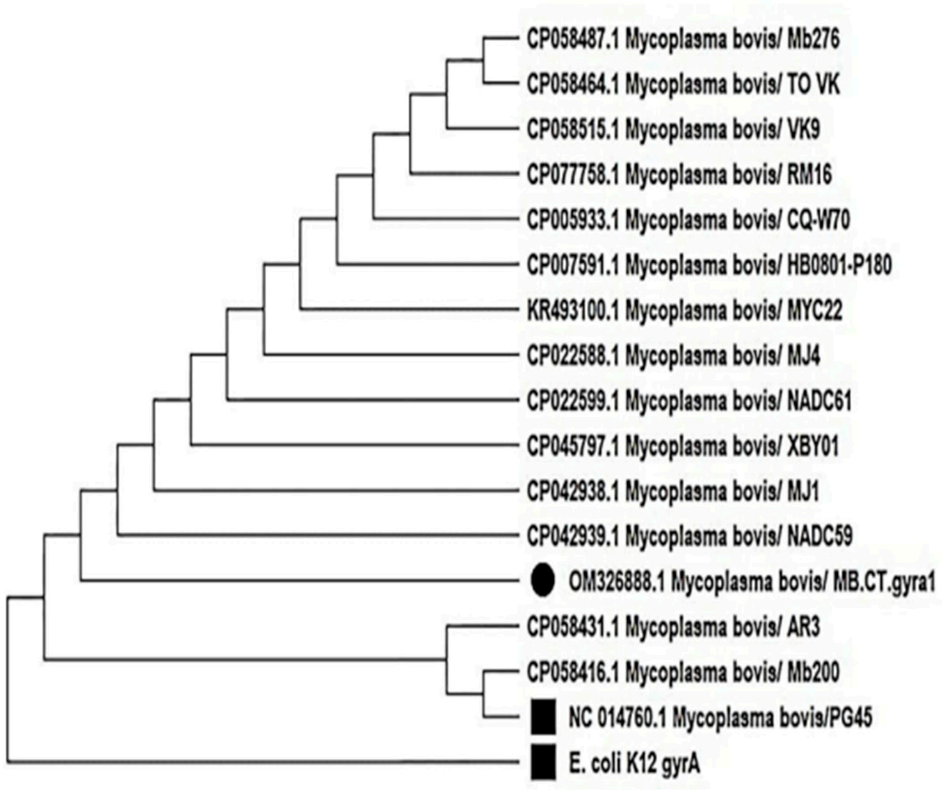

Supplementary Figure S1. Phylogenetic tree of the examined *M. bovis* isolate based on the *gyrA* sequence. ● Our examined *M. bovis* isolate, ■ Reference strains; *E. coli* K12 and *M. bovis* PG45 (control negative; enrofloxacin sensitive).

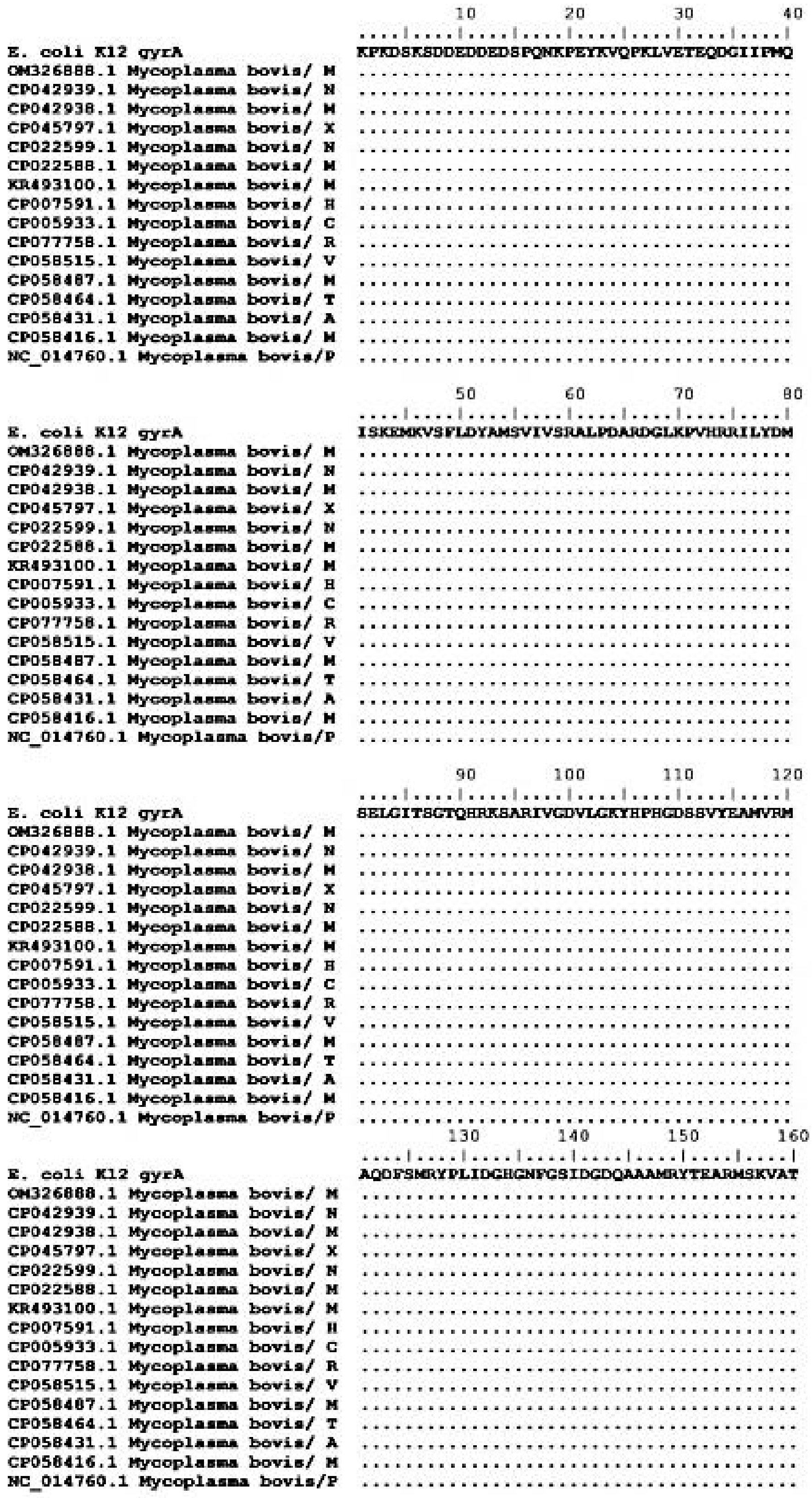

Supplementary Figure S2. Amino acid sequence alignments of the *gyrA* gene of the examined *M. bovis* isolate. The sequences are indicated by their accession numbers.

Seq->	E. coli K12 gyrA	OM326888.1 Mycoplasma bovis/ MB-CT.gyra1	CP042939.1 Mycoplasma bovis/ NADC59	CP042938.1 Mycoplasma bovis/ MJ1	CP045797.1 Mycoplasma bovis/ XBY01	CP022599.1 Mycoplasma bovis/ NADC61	CP022588.1 Mycoplasma bovis/ MJ4	KR493100.1 Mycoplasma bovis/ MYC22	CP007591.1 Mycoplasma bovis/ HB0801-P180	CP005933.1 Mycoplasma bovis/ CQ-W70	CP077758.1 Mycoplasma bovis/ RM16	CP058515.1 Mycoplasma bovis/ VK9	CP058487.1 Mycoplasma bovis/ Mb276	CP058464.1 Mycoplasma bovis/ TO VK	CP058431.1 Mycoplasma bovis/ AR3	CP058416.1 Mycoplasma bovis/ Mb200	NC_014760.1 Mycoplasma bovis/PG45
E. coli K12 gyrA	ID	100%	100%	100%	100%	100%	100%	100%	100%	100%	100%	100%	100%	100%	100%	100%	100%
OM326888.1 Mycoplasma bovis/ MB.CT.gyra1	100%	ID	100%	100%	100%	100%	100%	100%	100%	100%	100%	100%	100%	100%	100%	100%	100%
CP042939.1 Mycoplasma bovis/ NADC59	100%	100%	ID	100%	100%	100%	100%	100%	100%	100%	100%	100%	100%	100%	100%	100%	100%
CP042938.1 Mycoplasma bovis/ MJ1	100%	100%	100%	ID	100%	100%	100%	100%	100%	100%	100%	100%	100%	100%	100%	100%	100%
CP045797.1 Mycoplasma bovis/ XBY01	100%	100%	100%	100%	ID	100%	100%	100%	100%	100%	100%	100%	100%	100%	100%	100%	100%
CP022599.1 Mycoplasma bovis/ NADC61	100%	100%	100%	100%	100%	ID	100%	100%	100%	100%	100%	100%	100%	100%	100%	100%	100%
CP022588.1 Mycoplasma bovis/ MJ4	100%	100%	100%	100%	100%	100%	ID	100%	100%	100%	100%	100%	100%	100%	100%	100%	100%
KR493100.1 Mycoplasma bovis/ MYC22	100%	100%	100%	100%	100%	100%	100%	ID	100%	100%	100%	100%	100%	100%	100%	100%	100%
CP007591.1 Mycoplasma bovis/ HB0801-P180	100%	100%	100%	100%	100%	100%	100%	100%	ID	100%	100%	100%	100%	100%	100%	100%	100%
CP005933.1 Mycoplasma bovis/ CQ-W70	100%	100%	100%	100%	100%	100%	100%	100%	100%	ID	100%	100%	100%	100%	100%	100%	100%
CP077758.1 Mycoplasma bovis/ RM16	100%	100%	100%	100%	100%	100%	100%	100%	100%	100%	ID	100%	100%	100%	100%	100%	100%
CP058515.1 Mycoplasma bovis/ VK9	100%	100%	100%	100%	100%	100%	100%	100%	100%	100%	100%	ID	100%	100%	100%	100%	100%
CP058487.1 Mycoplasma bovis/ Mb276	100%	100%	100%	100%	100%	100%	100%	100%	100%	100%	100%	100%	ID	100%	100%	100%	100%
CP058464.1 Mycoplasma bovis/ TO VK	100%	100%	100%	100%	100%	100%	100%	100%	100%	100%	100%	100%	100%	ID	100%	100%	100%
CP058431.1 Mycoplasma bovis/ AR3	100%	100%	100%	100%	100%	100%	100%	100%	100%	100%	100%	100%	100%	100%	ID	100%	100%
CP058416.1 Mycoplasma bovis/ Mb200	100%	100%	100%	100%	100%	100%	100%	100%	100%	100%	100%	100%	100%	100%	100%	ID	100%
NC_014760.1 Mycoplasma bovis/PG45	100%	100%	100%	100%	100%	100%	100%	100%	100%	100%	100%	100%	100%	100%	100%	100%	ID

Supplementary Figure S3. Amino acid identity percentages between the *gyrA* genes of *E. coli* K12, *M. bovis gyrA* (OM326888), *M. bovis* PG45 (control negative; enrofloxacin sensitive), and other *M. bovis* isolates on GenBank. ID: identity.

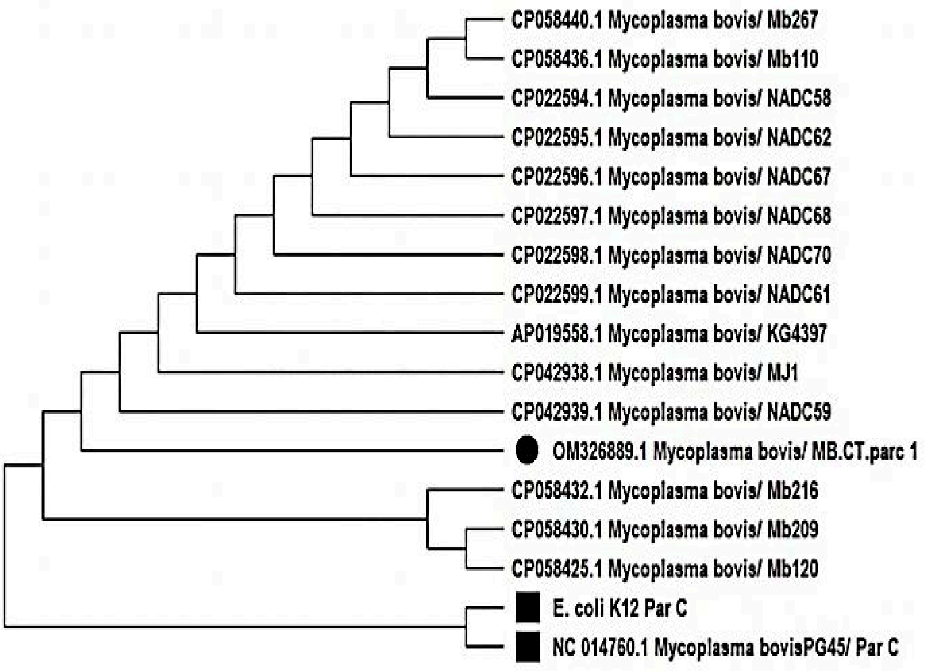

Supplementary Figure S4. Phylogenetic tree of the examined *M. bovis* isolate based on the *parC* sequence. • Our examined *M. bovis* isolate, ▪ Reference strains; *E. coli* K12 and *M. bovis* PG45 (control negative; enrofloxacin sensitive).

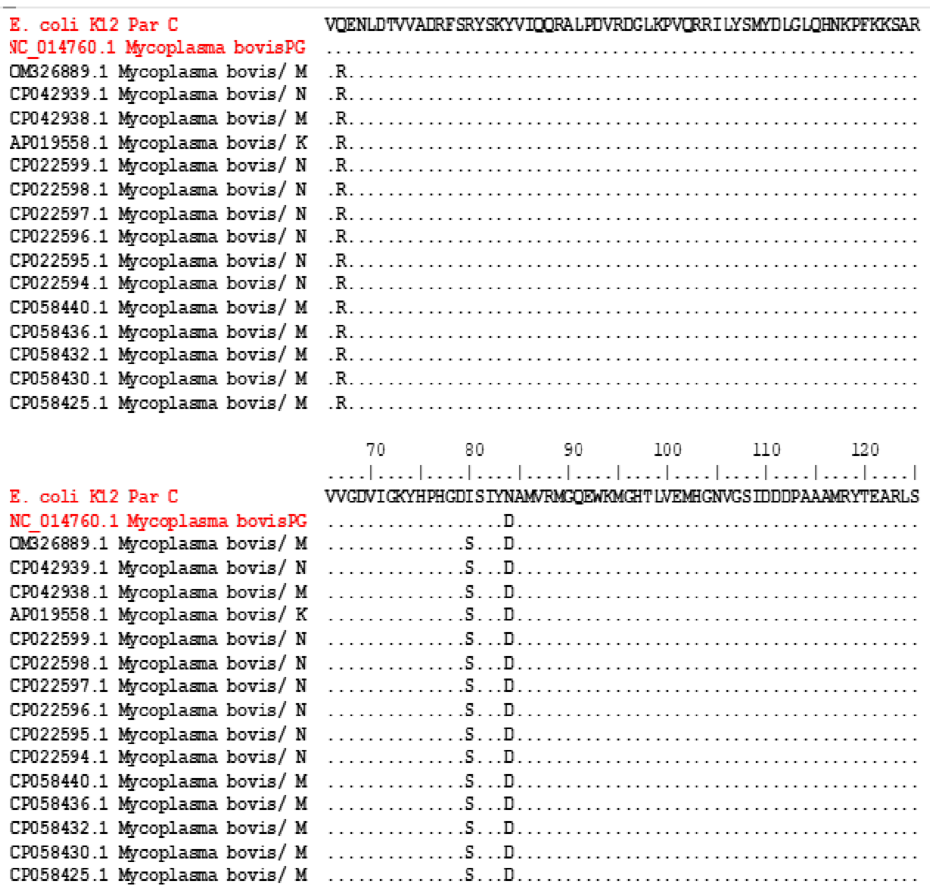

Supplementary Figure S5. Amino acid sequence alignments of the *parC* gene of the examined *M. bovis* isolate. The sequences are indicated by their accession numbers.

Seq->	E. coli K12 Par C	OM326889.1 Mycoplasma bovis/ MB.CT.parc 1	CP042939.1 Mycoplasma bovis/ NADC59	CP042938.1 Mycoplasma bovis/ MJ1	AP019558.1 Mycoplasma bovis/ KG4397	CP022599.1 Mycoplasma bovis/ NADC61	CP022598.1 Mycoplasma bovis/ NADC70	CP022597.1 Mycoplasma bovis/ NADC68	CP022596.1 Mycoplasma bovis/ NADC67	CP022595.1 Mycoplasma bovis/ NADC62	CP022594.1 Mycoplasma bovis/ NADC58	CP058440.1 Mycoplasma bovis/ Mb267	CP058436.1 Mycoplasma bovis/ Mb110	CP058432.1 Mycoplasma bovis/ Mb216	CP058430.1 Mycoplasma bovis/ Mb209	CP058425.1 Mycoplasma bovis/ Mb120	NC_014760.1 Mycoplasma bovisPG45/ Par C
E. coli K12 Par C	ID	98%	98%	98%	98%	98%	98%	98%	98%	98%	98%	98%	98%	98%	98%	98%	99%
OM326889.1 Mycoplasma bovis/ MB.CT.parc 1	98%	ID	100%	100%	100%	100%	100%	100%	100%	100%	100%	100%	100%	100%	100%	100%	99%
CP042939.1 Mycoplasma bovis/ NADC59	98%	100%	ID	100%	100%	100%	100%	100%	100%	100%	100%	100%	100%	100%	100%	100%	99%
CP042938.1 Mycoplasma bovis/ MJ1	98%	100%	100%	ID	100%	100%	100%	100%	100%	100%	100%	100%	100%	100%	100%	100%	99%
AP019558.1 Mycoplasma bovis/ KG4397	98%	100%	100%	100%	ID	100%	100%	100%	100%	100%	100%	100%	100%	100%	100%	100%	99%
CP022599.1 Mycoplasma bovis/ NADC61	98%	100%	100%	100%	100%	ID	100%	100%	100%	100%	100%	100%	100%	100%	100%	100%	99%
CP022598.1 Mycoplasma bovis/ NADC70	98%	100%	100%	100%	100%	100%	ID	100%	100%	100%	100%	100%	100%	100%	100%	100%	99%
CP022597.1 Mycoplasma bovis/ NADC68	98%	100%	100%	100%	100%	100%	100%	ID	100%	100%	100%	100%	100%	100%	100%	100%	99%
CP022596.1 Mycoplasma bovis/ NADC67	98%	100%	100%	100%	100%	100%	100%	100%	ID	100%	100%	100%	100%	100%	100%	100%	99%
CP022595.1 Mycoplasma bovis/ NADC62	98%	100%	100%	100%	100%	100%	100%	100%	100%	ID	100%	100%	100%	100%	100%	100%	99%
CP022594.1 Mycoplasma bovis/ NADC58	98%	100%	100%	100%	100%	100%	100%	100%	100%	100%	ID	100%	100%	100%	100%	100%	99%
CP058440.1 Mycoplasma bovis/ Mb267	98%	100%	100%	100%	100%	100%	100%	100%	100%	100%	100%	ID	100%	100%	100%	100%	99%
CP058436.1 Mycoplasma bovis/ Mb110	98%	100%	100%	100%	100%	100%	100%	100%	100%	100%	100%	100%	ID	100%	100%	100%	99%
CP058432.1 Mycoplasma bovis/ Mb216	98%	100%	100%	100%	100%	100%	100%	100%	100%	100%	100%	100%	100%	ID	100%	100%	99%
CP058430.1 Mycoplasma bovis/ Mb209	98%	100%	100%	100%	100%	100%	100%	100%	100%	100%	100%	100%	100%	100%	ID	100%	99%
CP058425.1 Mycoplasma bovis/ Mb120	98%	100%	100%	100%	100%	100%	100%	100%	100%	100%	100%	100%	100%	100%	100%	ID	99%
NC_014760.1 Mycoplasma bovisPG45/ Par C	99%	99%	99%	99%	99%	99%	99%	99%	99%	99%	99%	99%	99%	99%	99%	99%	ID

Supplementary Figure S6. Amino acid identity percentages between the *ParC* genes of *E. coli* K12, *M. bovis ParC* (OM326889), *M. bovis* PG45 (control negative; enrofloxacin sensitive), and other *M. bovis* isolates on Genebank. ID: identity.
